# Factors associated with anaemia among adolescent boys and girls 10–19 years old in Nepal

**DOI:** 10.1111/mcn.13013

**Published:** 2020-04-27

**Authors:** Nicole D. Ford, Ram Padarth Bichha, Kedar Raj Parajuli, Naveen Paudyal, Nira Joshi, Ralph D. Whitehead, Stanley Chitekwe, Zuguo Mei, Rafael Flores‐Ayala, Debendra P. Adhikari, Sanjay Rijal, Maria Elena Jefferds

**Affiliations:** ^1^ McKing Consulting Corporation Fairfax Virginia; ^2^ Nutrition Branch, Division of Nutrition, Physical Activity, and Obesity United States Centers for Disease Control and Prevention Atlanta Georgia; ^3^ Nepal Ministry of Health and Population Kathmandu Nepal; ^4^ Nutrition Section United Nations Children's Fund (UNICEF) Kathmandu Nepal; ^5^ New ERA Kathmandu Nepal; ^6^ United States Agency for International Development (USAID) Kathmandu Nepal

**Keywords:** adolescent, anaemia, iron, iron deficiency Anaemia, micronutrient malnutrition, Nepal, vitamin A

## Abstract

We used data from the 2016 Nepal National Micronutrient Status Survey to evaluate factors associated with anaemia (World Health Organization cut‐points using altitude‐ and smoking‐adjusted haemoglobin [Hb]) among nationally representative samples of adolescents 10–19 years. Hb, biomarkers of micronutrients, infection and inflammation were assessed from venous blood. Sociodemographic and household characteristics, dietary diversity, pica and recent morbidity were ascertained by interview. We explored bivariate relationships between candidate predictors and anaemia among boys (*N* = 967) and girls (*N* = 1,680). Candidate predictors with *P* < 0.05 in bivariate analyses were included in sex‐specific multivariable logistic regression models. Anaemia prevalence was 20.6% (95% confidence interval [CI] [17.1, 24.1]) among girls and 10.9% (95% CI [8.2, 13.6]) among boys. Among girls, living in the Mountain and Hill ecological zones relative to the Terai (adjusted odds ratio [AOR] 0.28, 95% CI [0.15, 0.52] and AOR 0.42, 95% CI [0.25, 0.73], respectively), ln ferritin (μg/L) (AOR 0.53, 95% CI [0.42, 0.68]) and ln retinol binding protein (RBP) (μmol/L) (AOR 0.08, 95% CI [0.04, 0.16]) were associated with reduced anaemia odds. Older age (age in years AOR 1.19, 95% CI [1.12, 1.27]) and Janajati ethnicity relative to the Muslim ethnicity (AOR 3.04, 95% CI [1.10, 8.36]) were associated with higher anaemia odds. Among boys, ln RBP [μmol/L] (AOR 0.25, 95% CI [0.10, 0.65]) and having consumed flesh foods (AOR 0.57, 95% CI [0.33, 0.99]) were associated with lower anaemia odds. Open defecation (AOR 2.36, 95% CI [1.15, 4.84]) and ln transferrin receptor [mg/L] (AOR 3.21, 95% CI [1.25, 8.23]) were associated with increased anaemia odds. Anaemia among adolescents might be addressed through effective public health policy and programs targeting micronutrient status, diet and sanitation.

**Key messages**

- Among adolescent girls, younger age (10–14 years vs. 15–19 years), residing in the Mountain or Hill ecological zones relative to the Terai ecological zone, serum ferritin and serum RBP were associated with reduced odds of anaemia, while belonging to the Janajati ethnicity relative to the Muslim ethnicity was associated with increased odds of anaemia.
- Among adolescent boys, serum RBP and having consumed flesh, organ or blood‐based foods were associated with reduced odds of anaemia, while open defecation and ln serum transferrin receptor were associated with increased odds of anaemia.
- A combination of effectively implemented strategies might reduce anaemia among adolescents 10–19 years in Nepal by addressing micronutrient status, diet and sanitation.
- Patterning of nonmodifiable factors, such as age, might explain differential success in reducing anaemia and might help inform program planning.

## 1 INTRODUCTION

Adolescence is an important developmental period. Deficits in this sensitive window can affect health in adulthood and that of future offspring (Sawyer et al., 2012). Globally, the prevalence of anaemia among adolescents is unknown. The World Health Organization (WHO) estimates that more than 50% of girls 12–15 years in South East Asia have anaemia; however, no estimates are available for boys (World Health Organization [WHO] Regional Office for South‐East Asia, 2011). Worldwide, iron‐deficiency anaemia is the leading contributor to disability‐adjusted life years among adolescents (WHO, 2015) and is associated with poor linear growth, delayed menarche and sexual development and reduced work capacity (Delisle and WHO, 2005). For adolescent girls and women, entering pregnancy with anaemia is also associated with poor maternal health and birth outcomes (Delisle and WHO, 2005).

Historically, Nepal has had high prevalence of anaemia. Although national‐level time trends are not available for adolescents 10–19 years, anaemia prevalence increased among adolescent girls 15–19 years from 39.0% in 2006 to 43.6% in 2016 according to the Demographic and Health Surveys (Ministry of Health, New ERA, ICF 2017). Other studies, some subnational and nonpopulation based, among adolescent girls in Nepal have reported anaemia prevalence values ranging from 38 to 69% (Baral & Onta, 2009; Chalise et al., 2018; Dubey et al., 2013; Kanodia, Bhatta, Sing, Bhatta, & Shah, 2016; Limbu et al., 2017; Shah & Gupta, 2002; Sinha, Karki, & Karna, 2012; Tiwari & Seshadri, 2000). To our knowledge, only three studies, two of which were subnational, have evaluated anaemia prevalence among adolescent boys in Nepal, which reported prevalence values ranging from 24 to 52% (Baral & Onta, 2009; Chalise et al., 2018; Sinha et al., 2012).

To develop effective, evidence‐based public health programming to address anaemia, it is critical to understand its context‐specific determinants. Although iron deficiency is the primary cause of anaemia worldwide (Ezzati, Lopez, Rodgers, & Murray, 2004), other biological factors contribute to anaemia including other micronutrient deficiencies, infection and inflammation. Intermediate causes of anaemia, such as dietary intake, influence biological determinants, while socio‐economic and other characteristics underlay many of the intermediate causes of anaemia.

Regionally, factors contributing to anaemia vary by population, highlighting the need to better understand context‐specific causes. Among women of reproductive age in rural Bangladesh, thalassemia, underweight and groundwater iron intake were associated with anaemia odds (Merrill et al., 2012). A nationally representative study of women of reproductive age in Cambodia found no factors significantly associated with anaemia in multivariable models (Wieringa et al., 2016). Little is known about context‐specific determinants of anaemia among adolescents in Nepal. Existing studies in Nepal have explored bivariate relationships between anaemia and iron status, dietary indicators or sociodemographic characteristics among adolescent girls (Chalise et al., 2018; Kanodia et al., 2016; Limbu et al., 2017; Shah & Gupta, 2002; Tiwari & Seshadri, 2000); however, to our knowledge, none have examined biomarkers of infection, inflammation and micronutrient status. Additionally, only one study has conducted multivariable modelling and included adolescent boys (Chalise et al., 2018).

Nepal conducted the Nepal National Micronutrient Status Survey (NNMSS) in 2016 to inform programmatic decision‐making. The NNMSS collected data on micronutrient status and many known potential causes of anaemia (Ministry of Health et al., 2018). We used nationally representative NNMSS data to identify factors associated with anaemia among nonpregnant adolescent girls and adolescent boys 10–19 years.

## 2 METHODS

### 2.1 Study population

New ERA, with support from the Ministry of Health and Population (MoHP) of Nepal, US Agency for International Development, United Nations Children's Fund Nepal and the US Centers for Disease Control and Prevention (CDC), implemented the 2016 NNMSS. Using stratified multistage cluster sampling without replacement, we selected 180 clusters from 15 strata using probability proportional to size. Using systematic random sampling, we then selected 24 households in each cluster (*N* = 4,320). After enumerating all adolescents 10–19 years in the selected households, we selected 6 boys and 12 girls from each cluster at random. Sample sizes were calculated with the primary objective of estimating anaemia prevalence at the national, ecological zone and developmental region levels, assuming anaemia prevalence values of 25% for boys and 39% for girls based on prevalence among adolescent girls 15–19 years in the 2011 Demographic and Health Surveys, a precision of ±3.5% nationally, a design effect of 2.25, a household response rate of 95% and an individual response rate of 90% (Ministry of Health and Population‐MOHP/Nepal et al., 2012). The multivariable analysis on the aetiology of anaemia among adolescents was a secondary objective. The NNMSS Report provides more details about the survey, populations and sampling strategy (Ministry of Health et al., 2018).

Census projections overestimated adolescent girls 10–19 years as a percentage of the total population. As a result, only 1,886 adolescent girls lived in the sampled households in the sampled clusters compared with the 2,160 planned for data collection. Of those, 1,850 were nonpregnant, consented to participate and were interviewed in the selected clusters (98.1%). We excluded girls with missing or invalid values for haemoglobin (Hb; *n* = 10), blood‐based indictors (*n* = 8 missing; *n* = 29 with equivocal results on *Helicobacter pylori* rapid test), anthropometry (*n* = 117) and questionnaire‐based data (*n* = 4). We additionally excluded *n* = 2 girls with biologically implausible body mass index (BMI) *Z* scores, per WHO guidance (de Onis et al., 2007) for a final analytic sample of 1,680 girls (89.1% of sampled girls). Among those who consented to participate, with respect to major sociodemographic characteristics, girls who were excluded from the analytic sample were on significantly older (17.9 years vs. 13.9 years) and more likely to be married than those who were included (Table S1).

Of the 1,080 adolescent boys planned for data collection, 1,040 lived in the selected clusters (96.3%). Of those, 1,025 consented to participate and were interviewed (98.6%). We excluded boys with missing or invalid values for Hb (*n* = 2), blood‐based indicators (*n* = 11), anthropometry (*n* = 39 missing; *n* = 3 with biologically implausible BMI Z scores) and questionnaire‐based indicators (*n* = 3) for a final analytic sample of 967 boys (94.3% of sampled boys). Among those who consented to participate, boys who were excluded from the analytic sample were significantly older (17.0 years vs. 13.9 years) and more likely to be married than those who were included (Table S1).

The Nepal Health Research Council granted ethical approval for the study. Adolescents aged 10–17 years provided oral assent for interview and biological data collection, and their legal guardians or parents provided signed informed consent. Adolescents aged 18 and older provided signed informed consent.

### 2.2 Data collection

The field survey team participated in an intensive 12‐day training conducted by core survey team members from New ERA and CDC that included classroom instruction, demonstrations, role play and mock interviews. Enumerators had anthropometry standardization exercises on live participants, comparing measurements to experts'. Phlebotomists were given practical examinations on blood draw and field testing samples. Laboratory technicians processed the samples the phlebotomists collected to standardize technique and practice proper sample storage. Trainees who performed poorly during these practical examinations were not retained for the field work. The teams were also deployed for a 3‐day pilot to test survey tools and field procedures.

#### 2.2.1 Anthropometry

Weight with one layer of light clothing was measured to the nearest 100 g using an electronic SECA digital scale. Standing height was measured without shoes to the nearest 0.1 cm using a Shorr‐Board. Enumerators validated the calibration of their anthropometry equipment daily.

#### 2.2.2 Biological specimens

Following standard procedures, trained phlebotomists collected venous blood samples at the time of interview at the household to assess micronutrient, infection and inflammation status. Technicians analysed Hb (HemoCue® Hb 301 analyser), *H. pylori* (QuickVue™ *H. pylori* Test rapid test kit) and malaria (CareStart™ malaria antigen combo rapid test kit for *Plasmodium falciparum* and *Plasmodium vivax* ) in the households. Phlebotomists validated the calibration of their HemoCue daily using standard reference materials. Laboratory technicians processed blood specimens at a lab station in each cluster before transport to the National Public Health Laboratory, maintaining cold chain. Protocols on quality assurance were adhered to as outlined in the laboratory manual. Plasma and serum samples were stored in −86°C freezers until analysis.

C‐reactive protein (CRP), ɑ‐1‐acid glycoprotein (AGP), serum ferritin, transferrin receptor (sTfR) and retinol binding protein (RBP) were measured using a sandwich enzyme‐linked immunosorbent assay (Erhardt, Estes, Pfieffer, Biesalski, & Craft, 2004). For girls only, red blood cell folate was analysed using a microbiological assay (Pfeiffer et al., 2011). All laboratories conducting biological analyses were required to follow quality control procedures and participate and have acceptable performance in CDC's external quality assurance program—VITAL‐EQA.

#### 2.2.3 Sociodemographic, health and other questionnaire data

In household questionnaires, the head of household or another adult respondent provided information about sociodemographic characteristics, and housing, water and sanitation characteristics and food security in enumerator‐administered interviews. Household food security was ascertained using a nine‐item questionnaire about access to adequate and preferred foods (Coates, Swindale, & Billinsky, 2007). Adolescents provided information about marital status, schooling, reproductive history (girls only), consumption of foods from 10 food groups (grains, legumes, nuts, dairy, flesh foods, eggs, green leafy vegetables, vitamin A‐rich fruits and vegetable, other fruits, and other vegetables) and tea, micronutrient supplement intake, pica, recent morbidity, and receipt of deworming tablets in enumerator‐administered interviews using gender‐specific questionnaires.

### 2.3 Variable specification

#### 2.3.1 Anaemia

We defined anaemia as altitude‐ and smoking‐adjusted Hb <11.5 g/dL for boys and girls 10–11 years, Hb <12.0 g/dL for boys 12–14 years and girls 12–19 years and Hb <13.0 for boys 15–19 years (WHO^a^, 2017). Anaemia severity was classified as mild (adjusted Hb 11.0–11.4 g/dL for boys and girls 10–11 years, Hb 11.0–11.9 g/dL for boys 12–14 years and girls 12–19 years and Hb 11.0–12.9 g/dL for boys 15–19 years), moderate (adjusted Hb 8.0–10.9 g/dL) and severe (adjusted Hb <8.0 g/dL; WHO^a^, 2017).

#### 2.3.2 Anthropometry

We calculated BMI‐for‐age *Z* scores (BMIZ) using the WHO growth reference for school‐aged children and adolescents. BMIZ was classified as underweight (BMIZ < −2 *SD*), normal weight (BMIZ ≥ −2 *SD* and ≤1 *SD*) and overweight (BMIZ >1 *SD*; de Onis et al., 2007).

#### 2.3.3 Biomarkers of nutrition status

To correct for inflammation's influence on biomarkers of iron status, we regression‐adjusted ferritin and sTfR to a pooled country reference using CRP and AGP (ferritin) or AGP only (sTfR; Namaste, Aaron, Varadhan, Peerson, & Suchdev, 2017). Iron deficiency by ferritin was defined as adjusted ferritin <15.0 μg/L (WHO^a^, 2017). We defined iron‐deficiency anaemia as adjusted Hb <11.5 g/dL and adjusted ferritin <15.0 μg/L for boys and girls 10–11 years, adjusted Hb <12.0 g/dL and adjusted ferritin <15.0 μg/L among boys 12–14 years and girls 12–19 years and adjusted Hb <13.0 g/dL and adjusted ferritin <15.0 μg/L for boys 15–19 years. Iron deficiency by sTfR was defined as adjusted sTfR >8.3 μg/L (WHO^a^, 2017). We defined vitamin A deficiency as RBP <0.64 μmol/L. To find the RBP cut‐point equivalent of retinol <0.70 μmol/L (WHO, 1996) among adolescents, we regressed RBP on retinol in an NNMSS subsample of 100 women 15–49 years for whom serum retinol was assessed using high‐performance liquid chromatography from the same blood draw as RBP. For girls, risk of folate deficiency was classified as red blood cell folate <305.0 nmol/L based on risk of megaloblastic anemia (Institute of Medicine, 1998).

#### 2.3.4 Infection and inflammation

CRP and AGP were included as continuous variables. We included malaria, *H. pylori* , and fever, diarrhoea and cough during the 2 weeks preceding the survey as binary variables (yes/no).

#### 2.3.5 Dietary intake

We defined pica as any consumption of clay, earth, termite mounds, ice, uncooked rice or starch during the 7 days before the survey. Food and Agriculture Organization (FAO)'s Minimum Dietary Diversity for Women of Reproductive Age (MDD‐W) indicator was classified as intake from five or more of 10 food groups the day preceding the survey (FAO and FHI 360, 2016). We included consumption of flesh, organ or blood‐based foods, legumes, green leafy vegetables, vitamin A‐rich fruits and vegetables and tea the day preceding the survey as binary variables (yes/no).

#### 2.3.6 Reproductive and other health variables

We included intake of any micronutrient supplements (multivitamin, vitamin A, iron, folic acid and/or zinc) the week preceding the survey, intake of iron‐folic acid (IFA) tablets during the 6 months preceding the survey and receipt of deworming tablets during the 6 months preceding the survey as binary variables (yes/no). For girls, lactation status and giving birth during the 5 years preceding the survey were included as binary variables (yes/no).

#### 2.3.7 Sociodemographic variables

Age in years was included as a continuous variable. Ethnicity was classified according to the Government of Nepal Central Bureau of Statistics: Brahmin/Chettri, Dalit, Janajati, other Terai ethnicities (including Terai/Madhesi but not including Terai or Madhesi Brahmin or Chettri), Newar and Muslim (Government of Nepal Central Bureau of Statistics, 2014). We categorized marital status as married or cohabitating as married vs. other. Schooling was categorized as never vs. ever attended school. We defined household location according to Nepal administrative classifications for rurality (rural vs. urban) and ecological zone (Mountain, Hill or Terai [Plains]). Using principal components analysis of housing characteristics and assets, we created a household wealth score and divided it into tertiles. We calculated a categorical indicator of household food insecurity (access), in accordance with the Household Food Insecurity Access Scale Indicator Guide, which classifies households into four levels of food insecurity: food secure, mildly food insecure, moderately food insecure and severely food insecure (Coates et al., 2007). We classified households as increasingly food insecure when they responded affirmatively to more severe conditions (e.g. no food to eat of any kind in the household because of lack of resources to get food) or experience of those conditions more frequently. Unimproved water source was defined as any source other than piped water, tube well borehole, protected well or spring, stone tap, rainwater or bottled water (WHO and UNICEF, 2017). We included unimproved water source, open defecation and earth floor as binary variables (yes/no) and household food security as a multilevel categorical variable.

### 2.4 Statistical methods

We log transformed nonnormally distributed continuous variables. We assessed bivariate relationships between candidate predictors and anaemia status by sex using Rao‐Scott chi square tests for categorical variables and linear contrast tests for continuous variables. Variables with multiple categories (e.g. household food security) were tested as a group. All candidate predictors of anaemia with *P* < 0.05 in bivariate analyses were included in the sex‐specific multivariable logistic regression models. To identity collinearity, we used eigenvalues <0.01 and conditionality index >30.

All analyses accounted for complex sampling design and were analysed using SAS v.9.4 (SAS Institute Inc., Cary, North Carolina). We set statistical significance at two‐sided *P* < 0.05.

## 3 RESULTS

### 3.1 Girls

In total, 20.6% (95% confidence interval [CI] [17.1, 24.1]) of girls had anaemia of which 68.3% (95% CI [61.7, 74.9]) were mild cases and 30.9% (95% CI [24.6, 37.2]) were moderate cases (Table 1). One third of girls with anaemia (33.2%, 95% CI [26.7, 39.7]) had iron deficiency by ferritin status.

**Table 1:** Selected sociodemographic and health characteristics of nonpregnant adolescent girls 10–19 years by anaemia status, Nepal National Micronutrient Status Survey, Nepal, 2016 (*N* = 1,680)^a^

Sociodemographic, health, and dietary characteristics	Anaemia (*N* = 317, 20.6% [95% CI 17.1, 24.1])		No Anaemia (N = 1,363, 79.4% [95% CI 75.9, 82.9])			Total (N = 1,680)	
	*n*		*n*		*P* ^b^	*n*	
Sociodemographic characteristics							
Age, years	317	14.5 (14.2, 14.7)	1,363	13.8 (13.6, 13.9)	<0.0001	1,680	13.9 (13.8, 14.1)
Lactating (%)	13	3.9 (1.5, 6.3)	34	2.5 (1.6, 3.4)	0.2	47	2.8 (1.9, 3.6)
Gave birth in last 5 years (%)	13	3.9 (1.5, 6.3)	37	2.6 (1.7, 3.5)	0.3	50	2.9 (2.0, 3.7)
Married/cohabitating (%)	28	9.5 (5.2, 13.7)	95	7.3 (5.6, 9.0)	0.2	123	7.7 (5.9, 9.5)
Rurality (%)					0.7		
Rural	274	89.6 (82.8, 96.4)	1,217	90.5 (85.8, 95.2)		1,491	90.3 (85.5, 95.2)
Urban	43	10.4 (3.6, 17.2)	146	9.5 (4.8, 14.2)		189	9.7 (4.8, 14.5)
Ecological zone (%)					<0.0001		
Mountain	23	3.2 (1.4, 5.0)	242	8.7 (7.0, 10.3)		265	7.6 (6.2, 8.9)
Hill	95	28.7 (19.8, 37.6)	612	47.3 (43.0, 51.6)		707	43.5 (39.5, 47.4)
Terai	199	68.1 (59.1, 77.1)	509	44.0 (39.8, 48.2)		708	49.0 (45.1, 52.8)
Household wealth tertile					0.2		
Poorest	95	27.9 (20.0, 35.8)	543	34.4 (29.5, 39.3)		638	33.0 (28.2, 37.9)
Middle	119	37.6 (31.5, 43.6)	440	34.7 (30.8, 38.6)		559	35.3 (31.6, 39.0)
Wealthiest	103	34.5 (25.5, 43.5)	380	30.9 (25.3, 36.4)		483	31.6 (26.1, 37.1)
Ethnicity (%)					0.02		
Brahmin or Chettri	96	24.1 (15.3, 32.9)	555	35.9 (29.6, 42.2)		651	33.5 (27.6, 39.4)
Dalit	54	15.3 (8.9, 21.8)	237	16.8 (12.5, 21.2)		291	16.5 (12.3, 20.8)
Janajati	122	40.0 (29.6, 50.4)	420	31.1 (24.5, 37.7)		542	32.9 (26.2, 39.7)
Other Terai ethnicities^c^	34	16.5 (8.7, 24.3)	79	10.3 (5.8, 14.8)		113	11.5 (6.9, 16.2)
Newar	4	1.5 (0.0, 4.0)^d^	45	3.7 (1.7, 5.6)		49	3.2 (1.6, 4.9)
Muslim	7	2.6 (0.2, 4.9)^d^	27	2.3 (0.4, 4.1)		34	2.3 (0.6, 4.0)
Never attended school (%)	16	7.8 (1.2, 14.3)	46	5.1 (2.9, 7.2)	0.2	62	5.6 (2.7, 8.5)
Unimproved water source^e^ (%)	10	3.3 (0.0, 7.7)	49	4.8 (2.1, 7.6)	0.5	59	4.5 (1.7, 7.3)
Open defecation (%)	55	27.7 (16.2, 39.2)	147	15.9 (11.2, 20.5)	0.002	202	18.3 (12.7, 23.9)
Earth floor (%)	225	68.4 (60.3, 76.5)	978	69.8 (64.1, 75.5)	0.7	1,203	69.5 (64.1, 74.9)
Household food insecurity^f^ (%)					0.3		
Food secure	201	59.0 (49.3, 68.8)	811	57.2 (52.2, 62.2)		1,012	57.6 (52.4, 62.8)
Mild food insecurity	64	25.3 (18.6, 31.9)	281	21.9 (18.4, 25.4)		345	22.6 (19.4, 25.8)
Moderate food insecurity	25	8.3 (2.9, 13.8)	157	13.2 (10.2, 16.3)		182	12.2 (9.3, 15.2)
Severe food insecurity	27	7.3 (4.1, 10.6)	114	7.7 (5.4, 9.9)		141	7.6 (5.4, 9.8)
Health characteristics							
Haemoglobin^g^ (g/dL)	317	11.0 (10.9, 11.2)	1,363	13.1 (13.0, 13.13)	<0.0001	1,680	12.6 (12.5, 12.7)
Anaemia severity^h^							
No anaemia	0	‐	1,363	‐		1,363	79.4 (75.9, 82.9)
Mild	212	68.3 (61.7, 74.9)	0	‐		212	14.1 (11.2, 16.9)
Moderate	103	30.9 (24.6, 37.2)	0	‐		103	6.4 (4.8, 7.9)
Severe	2	0.8 (0.0, 2.0)^d^	0	‐		2	0.2 (0.0, 0.4)^4^
Anthropometry^i^ (%)					0.9		
Underweight	43	13.0 (9.0, 17.1)	172	14.2 (11.3, 17.1)		215	14.0 (11.5, 16.4)
Normal weight	263	82.7 (78.3, 87.2)	1,124	81.5 (78.2, 84.8)		1,387	81.8 (79.1, 84.5)
Overweight/obesity	11	4.2 (1.3, 7.2)	67	4.3 (2.9, 5.7)		78	4.3 (3.0, 5.5)
Two week morbidity recall (%)							
Fever	52	17.6 (11.8, 23.4)	202	14.2 (11.5, 16.9)	0.3	254	14.9 (12.4, 17.4)
Cough	58	18.4 (13.6, 23.3)	268	18.8 (15.9, 21.7)	0.9	326	18.7 (16.4, 21.1)
Diarrhoea	24	7.4 (4.0, 10.9)	119	9.0 (6.7, 11.4)	0.4	143	8.7 (6.5, 10.8)
CRP (mg/L)	317	0.21 (0.17, 0.26)	1,363	0.22 (0.19, 0.24)	0.9	1,680	0.21 (0.19, 0.24)
AGP (g/L)	317	0.56 (0.53, 0.60)	1,363	0.54 (0.52, 0.56)	0.2	1,680	0.55 (0.53, 0.56)
Malaria (%)	0	‐	0	‐	‐	0	‐
Helicobacter pylori (%)	55	16.3 (11.8, 20.8)	214	15.6 (13.3, 18.0)	0.8	269	15.8 (13.8, 17.8)
Received deworming^j^ (%)	199	53.3 (45.8, 60.8)	855	55.5 (50.9, 60.1)	0.6	1,054	55.0 (50.8, 59.3)
Micronutrient status							
Serum ferritin^k^ (μg/L)	317	21.6 (18.6, 25.0)	1,363	30.5 (29.0, 32.1)	<0.0001	1,680	28.4 (26.8, 30.1)
Iron deficiency by ferritin^l^ (%)	112	33.2 (26.7, 39.7)	183	13.3 (10.8, 15.9)	<0.0001	295	17.4 (14.8, 20.1)
Serum sTfR^k^ (mg/L)	317	7.8 (7.2, 8.4)	1,363	5.8 (5.7, 5.9)	<0.0001	1,680	6.1 (6.0, 6.3)
Iron deficiency by sTfR^m^ (%)	120	35.4 (28.8, 42.0)	116	8.5 (6.6, 10.4)	<0.0001	236	14.0 (11.9, 16.1)
Serum RBP (μmol/L)	317	0.98 (0.95, 1.02)	1,363	1.14 (1.11, 1.16)	<0.0001	1,680	1.10 (1.08, 1.12)
Vitamin A deficiency^n^ (%)	12	5.6 (1.4, 9.8)	18	1.9 (0.8, 3.1)	0.02	30	2.7 (1.4, 4.0)
RBC folate (nmol/L)	317	441.2 (413.2, 471.2)	1,363	457.1 (440.0, 474.8)	0.3	1,680	453.8 (437.9, 470.2)
Risk of folate deficiency^o^ (%)	63	19.1 (14.2, 24.0)	241	15.5 (12.9, 18.1)	0.2	304	16.2 (13.8, 18.6)
Dietary and supplement intake							
Prior day food consumption (%)							
Flesh, organ or blood‐based foods	226	70.1 (62.4, 77.8)	982	71.8 (67.7, 75.8)	0.7	1,208	71.4 (67.5, 75.4)
Legumes	85	28.5 (22.1, 34.9)	405	31.1 (27.3, 34.8)	0.4	490	30.5 (26.9, 34.2)
Green, leafy vegetables	169	50.9 (43.9, 57.9)	746	54.3 (50.2, 58.3)	0.4	915	53.6 (49.8, 57.3)
Vitamin A‐rich fruits or vegetables	250	77.8 (68.8, 86.9)	1,125	81.7 (77.7, 85.7)	0.3	1,375	80.9 (76.7, 85.1)
Tea or Tibetan tea	150	46.8 (37.6, 55.9)	730	50.7 (45.9, 55.5)	0.4	880	49.9 (45.1, 54.6)
Minimum dietary diversity^p^	145	44.0 (34.6, 53.3)	578	42.9 (38.9, 46.9)	0.8	723	43.1 (38.9, 47.3)
Pica (%)	65	12.8 (7.8, 17.7)	202	12.3 (9.7, 14.9)	0.9	267	12.4 (9.8, 15.0)
Consumed micronutrient supplement^q^ (%)	6	2.5 (0.3, 4.6)^d^	13	1.1 (0.5, 1.8)	0.07	19	1.4 (0.6, 2.2)
Consumed iron‐folic acid^j^ (%)	12	3.6 (1.0, 6.1)	18	1.2 (0.6, 1.9)	0.01	30	1.7 (1.0, 2.5)

Abbreviations: AGP: ɑ‐1 acid glycoprotein; BMI: body mass index; BMIZ: BMI‐for‐age *Z* scores; CI: confidence interval; CRP: C‐reactive protein; FAO: Food and Agriculture Organization; Hb: haemoglobin; HPLC: high‐performance liquid chromatography; HPLC: high‐performance liquid chromatography; NNMSS: Nepal National Micronutrient Status Survey; RBC: red blood cell; RBP: retinol binding protein; sTfR: transferrin receptor.

a Ns are unweighted. Values presented are geometric mean (95% CI) or percent (95% CI). All estimates account for weighting and complex sampling design. Anaemia defined as altitude‐ and smoking‐adjusted Hb <11.5 g/dL for girls 10–11 years and altitude‐ and smoking‐adjusted Hb <12.0 g/dL for girls 12–19 years (WHO^a^, 2017).

b *P* values calculated for Rao‐Scott chi square tests (categorical) and linear contrast tests (continuous).

c Other Terai ethnicities include Terai/Madhesi ethnicities not including Terai/Madhesi Brahmin/Chettri (Government of Nepal Central Bureau of Statistics, 2014).

d Interpret with caution. Estimates may be unstable due to small *n.*

e Water source based on self‐report. Unimproved water source defined as any source other than piped water, tubewell borehole, protected well or spring, stone tap, rainwater or bottle water (UNICEF & WHO, 2017).

f Household food insecurity was categorized according to the Household Food Insecurity Access Scale Indicator Guide (Coates, Swindale, & Billinksy, 2007).

g Haemoglobin adjusted for altitude and smoking (WHO^a^, 2017).

h Anaemia severity categorized as mild (adjusted Hb 11.0–11.4 g/dL for 10–11 years and adjusted Hb 11.0–11.9 g/dL for 12–19 years), moderate anaemia (adjusted Hb 8.0–10.9 g/dL) and severe (adjusted Hb < 8.0 g/dL; WHO^a^, 2017).

i Underweight defined as BMIZ < −2 *SD.* Normal weight defined as BMIZ ≥ −2 *SD* and BMIZ ≤1 *SD.* Overweight defined as BMIZ >1 *SD* (deOnis et al 2007).

j During the 6 months preceding the survey.

k Biomarker was regression‐adjusted to a pooled country reference to adjust for inflammation, using CRP and AGP (ferritin) or AGP only (sTfR; Namaste et al., 2017).

l Iron deficiency defined as inflammation‐adjusted serum ferritin <15.0 μg/L (WHO^a^, 2017).

m Iron deficiency by sTfR defined as inflammation‐adjusted serum sTfR >8.3 μg/L (WHO^a^, 2017).

n We defined vitamin A deficiency as RBP <0.64 μmol/L. To find the RBP cut‐point equivalent of retinol <0.70 μmol/L (WHO, 1996) among adolescents, we regressed RBP on retinol in an NNMSS subsample of 100 women 15–49 years for whom serum retinol was assessed using HPLC from the same blood draw as RBP.

o Folate cutoff based on the risk of megaloblastic anaemia defined as RBC folate <305.0 nmol/L (Institute of Medicine 1998).

p Minimum dietary diversity defined as intake from ≥5 of the 10 main food groups (grains, legumes, nuts, dairy, flesh foods, eggs, green leafy vegetables, vitamin A‐rich fruits and vegetables, other fruits and other vegetables) the day preceding the survey based on FAO recommendations for minimum dietary diversity for women of reproductive age (MDD‐W) (FAO and FHI 360, 2016).

q Reported micronutrient supplement intake includes multivitamin, vitamin A, iron tablets or syrup, folic acid and/or zinc tablets consumed the week preceding the survey.

Results of the bivariate analyses are presented in Table S2. Candidate predictors (*P* < 0.05 in bivariate analyses) in the multivariable model included both potentially modifiable factors (open defecation, micronutrient status [ferritin, sTfR, RBP] and IFA intake) and nonmodifiable factors (age, ecological zone and ethnicity). We found collinearity between ferritin and sTfR. Because ferritin is the WHO recommended indicator to assess iron status in populations (WHO^a^, 2017), we removed sTfR from the multivariable model.

In the multivariable model, only two potentially modifiable factors were associated with anaemia among adolescent girls (Table 2). Iron status (ln ferritin in μg/L) and vitamin A status (ln RBP in μmol/L) were both associated with reduced odds of anaemia (adjusted odds ratio [AOR] 0.53, 95% CI [0.42, 0.68] and 0.08, 95% CI [0.04, 0.16], respectively).

**Table 2:** Bivariate and multivariable logistic regression predicting anaemia among nonpregnant adolescent girls 10–19 years, Nepal National Micronutrient Status Survey, Nepal, 2016 (*n* = 1,680)^a^

Potential predictors of anaemia	Unadjusted odds ratio (95% CI)	Adjusted odds ratio (95% CI)	*P*
Age in years	1.11 (1.06, 1.16)	1.19 (1.12, 1.27)	<0.0001
Ecological zone (ref. Terai)			
Mountain	0.24 (0.12, 0.46)	0.28 (0.15, 0.52)	<0.0001
Hill	0.39 (0.24, 0.63)	0.42 (0.25, 0.73)	0.002
Ethnicity (ref. Muslim)			
Dalit	0.81 (0.28, 2.30)	1.36 (0.51, 3.65)	0.5
Janajati	1.14 (0.39, 3.38)	3.04 (1.10, 8.36)	0.03
Other Terai ethnicities^b^	1.43 (0.44, 4.67)	1.80 (0.61, 5.35)	0.3
Newar	0.36 (0.05, 2.76)	1.07 (0.13, 8.51)	0.9
Brahmin/Chettri	0.59 (0.20, 1.81)	1.57 (0.53, 4.68)	0.4
Open defecation	2.03 (1.26, 3.28)	1.31 (0.79, 2.17)	0.3
Consumed iron‐folic acid	2.97 (1.19, 7.44)	2.52 (0.97, 6.54)	0.06
Ln ferritin in μg/L^c^	0.51 (0.40, 0.66)	0.53 (0.42, 0.68)	<0.0001
Ln RBP in μmol/L	0.12 (0.07, 0.23)	0.08 (0.04, 0.16)	<0.0001

Abbreviations: AGP: ɑ‐1 acid glycoprotein; CI: confidence interval; CRP: C‐reactive protein; Hb: haemoglobin; RBP; retinol‐binding protein.

a Estimates are unadjusted and adjusted odds ratios and 95% confidence intervals from bivariate and multivariable logistic regression, respectively. All analyses account for weighting and complex sampling design. Anaemia defined as altitude‐ and smoking‐adjusted Hb <11.5 g/dL for girls 10–11 years and altitude‐ and smoking‐adjusted Hb <12.0 g/dL for girls 12–19 years (WHO^a^, 2017). Candidate predictors were those where *P* < 0.05 in bivariate analyses.

b Other Terai ethnicities include Terai/Madhesi ethnicities not including Terai/Madhesi Brahmin/Chettri (Government of Nepal Central Bureau of Statistics, 2014).

c Biomarker was regression‐adjusted to a pooled country reference to adjust for inflammation, using CRP and AGP (Namaste et al., 2017).

Age, ecological zone and ethnicity were nonmodifiable factors associated with anaemia. Age in years was associated with increased odds of anaemia (AOR 1.19, 95% CI [1.12, 1.27]). Compared with living in the Terai ecological zone, girls living in the Mountain and Hill ecological zones had lower odds of anaemia (AOR 0.28, 95% CI [0.15, 0.52] and AOR 0.42, 95% CI [0.25, 0.73], respectively). Girls in the Janajati ethnicity had 3.04 times higher odds of anaemia relative to girls from the Muslim ethnicity (95% CI [1.10, 8.36]).

### 3.2 Boys

In total, 10.9% (95% CI [8.2, 13.6]) of boys had anaemia of which 84.9% (95% CI [75.2, 94.5]) were mild cases (Table 3).

**Table 3:** Selected sociodemographic and health characteristics of adolescent boys 10–19 years by anaemia status, Nepal National Micronutrient Status Survey, Nepal, 2016 (*N* = 967)^a^

Sociodemographic, health, and dietary characteristics	Anaemia (*N* = 88, 10.9% [95% CI 8.2, 13.6])		No Anaemia (*N* = 879, 89.1% [95% CI 86.4, 91.8])			Total (*N* = 967)	
	*n*		*n*		*P* ^b^	*n*	
Sociodemographic characteristics							
Age, years	88	14.0 (13.4, 14.7)	879	13.9 (13.6, 14.1)	0.6	967	13.9 (13.7, 14.1)
Married/cohabitating (%)	3	5.0 (0.0, 10.8)^c^	21	2.1 (1.0, 3.3)	0.2	24	2.4 (1.3, 3.6)
Rurality(%)					0.1		
Rural	79	91.4 (83.6, 99.2)	751	85.2 (78.3, 92.2)		830	85.9 (79.1, 92.6)
Urban	9	8.6 (0.8, 16.4)^c^	128	14.8 (7.8, 21.7)		137	14.1 (7.4, 20.9)
Ecological zone (%)					<0.0001		
Mountain	6	2.4 (0.5, 4.3)^c^	142	7.4 (6.3, 8.4)		148	6.8 (5.9, 7.7)
Hill	33	29.7 (18.0, 41.4)	377	43.0 (39.1, 46.9)		410	41.6 (37.8, 45.3)
Terai	49	67.9 (56.1, 79.8)	360	49.6 (45.9, 53.4)		409	51.6 (47.9, 55.4)
Household wealth tertile					0.2		
Poorest	31	31.5 (17.1, 45.9)	286	25.4 (20.6, 30.1)		317	26.0 (21.2, 30.9)
Middle	34	41.8 (26.3, 57.4)	301	35.9 (31.0, 40.8)		335	36.6 (31.5, 41.6)
Wealthiest	23	26.7 (14.5, 38.8)	292	38.7 (32.1, 45.4)		315	37.4 (30.9, 43.9)
Ethnicity (%)					0.08		
Brahmin or Chettri	34	26.8 (14.9, 38.6)	375	36.2 (30.3, 42.0)		409	35.1 (29.4, 40.9)
Dalit	9	9.9 (1.8, 18.0)^c^	140	14.9 (10.4, 19.4)		149	14.4 (10.0, 18.7)
Janajati	33	41.7 (26.0, 57.3)	252	28.1 (22.4, 33.9)		285	29.6 (23.6, 35.6)
Other Terai ethnicities^d^	10	18.9 (6.3, 31.4)	58	12.9 (8.0, 17.8)		68	13.5 (8.4, 18.7)
Newar	1	1.4 (0.0, 4.3)^c^	33	4.6 (1.6, 7.6)		34	4.3 (1.6, 6.9)
Muslim	1	1.3 (0.0, 4.0)^c^	21	3.3 (1.1, 5.4)		22	3.1 (1.0, 5.2)
Never attended school (%)	2	3.8 (0.0, 9.6)^c^	12	1.8 (0.6, 3.0)	0.2	14	2.0 (0.5, 3.5)
Unimproved water source^e^ (%)	5	5.6 (0.0, 12.1)^c^	29	4.4 (1.4, 7.4)	0.7	34	4.5 (1.5, 7.6)
Open defecation (%)	15	33.3 (17.3, 49.2)	67	11.8 (7.8, 15.8)	<0.0001	82	14.2 (9.4, 19.0)
Earth floor (%)	67	75.5 (64.6, 86.5)	578	63.2 (57.3, 69.2)	0.03	645	64.6 (58.7, 70.5)
Household food insecurity^f^ (%)					0.3		
Food secure	53	58.1 (41.7, 74.5)	526	60.4 (55.7, 65.0)		579	60.1 (55.2, 65.1)
Mild food insecurity	22	24.2 (12.8, 35.5)	187	22.5 (18.7, 26.4)		209	22.7 (18.9, 26.5)
Moderate food insecurity	8	14.8 (0.3, 29.2)^c^	85	8.7 (5.9, 11.6)		93	9.4 (6.5, 12.3)
Severe food insecurity	5	2.9 (0.0, 5.9)^c^	81	8.3 (5.8, 10.9)		86	7.8 (5.5, 10.0)
Health characteristics							
Haemoglobin^g^ (g/dL)	88	11.8 (11.6, 12.0)	879	14.0 (13.9, 14.1)	<0.0001	967	13.7 (13.6, 13.8)
Anaemia severity^h^							
No anaemia	0	‐	879	‐		879	89.1 (86.4, 91.8)
Mild	75	84.9 (75.2, 94.5)	‐	‐		74	9.3 (7.0, 11.5)
Moderate	13	15.1 (5.5, 24.8)	‐	‐		13	1.7 (0.5, 2.8)
Severe	0	‐	‐	‐		0	‐
Anthropometry^i^ (%)					0.05		
Underweight	29	32.8 (20.2, 45.4)	184	22.5 (19.3, 25.8)		213	23.7 (20.2, 27.1)
Normal weight	58	66.3 (53.6, 78.9)	667	72.3 (68.8, 75.7)		725	71.6 (68.1, 75.1)
Overweight/obesity	1	1.0 (0.0, 2.9)^c^	28	5.2 (2.7, 7.8)		29	4.7 (2.5, 7.0)
Two‐week morbidity recall (%)							
Fever	14	13.7 (5.4, 22.1)	108	10.1 (7.5, 12.6)	0.4	122	10.5 (8.1, 12.9)
Cough	5	6.0 (0.0, 11.9)^c^	130	12.4 (9.7, 15.0)	0.1	135	11.7 (9.3, 14.1)
Diarrhoea	8	10.2 (2.2, 18.3)^c^	60	6.6 (4.6, 8.7)	0.4	68	7.0 (5.2, 8.9)
CRP (mg/L)	88	0.28 (0.16, 0.50)	879	0.23 (0.20, 0.27)	0.6	967	0.24 (0.21, 0.28)
AGP (g/L)	88	0.54 (0.48, 0.59)	879	0.52 (0.50, 0.53)	0.5	967	0.52 (0.50, 0.54)
Malaria (%)	0	‐	0	‐	‐	0	‐
Helicobacter pylori (%)	15	13.5 (5.8, 21.3)	129	13.2 (10.7, 15.6)	0.9	144	13.2 (10.7, 15.6)
Received deworming^j^ (%)	62	56.1 (41.9, 70.3)	540	53.2 (48.6, 57.8)	0.7	602	53.6 (48.9, 58.2)
Micronutrient status							
Serum ferritin^k^, μg/L	88	45.6 (36.2, 57.6)	879	43.9 (41.2, 46.7)	0.7	967	44.0 (41.3, 47.0)
Iron deficiency by ferritin^l^ (%)	7	8.6 (1.7, 15.4)^c^	29	4.5 (2.4, 6.6)	0.2	36	5.0 (2.9, 7.0)
Serum sTfR^k^ (mg/L)	88	7.4 (6.6, 8.4)	879	6.2 (6.0, 6.3)	0.004	967	6.3 (6.1, 6.5)
Iron deficiency by sTfR^m^ (%)	24	30.8 (16.4, 45.2)	86	10.2 (7.0, 13.4)	<0.0001	110	12.4 (9.3, 15.6)
Serum RBP (μmol/L)	88	1.01 (0.95, 1.07)	879	1.19 (1.16, 1.22)	<0.0001	967	1.16 (1.14, 1.19)
Vitamin A deficiency^n^ (%)	4	5.9 (0.0, 12.1)^c^	8	1.1 (0.2, 2.1)^c^	<0.0001	12	1.6 (0.4, 2.9)
Dietary and supplement intake							
Prior day food consumption (%)							
Flesh, organ, or blood‐based foods	67	80.9 (71.4, 90.4)	604	68.4 (64.3, 72.5)	0.02	671	69.8 (65.7, 73.8)
Legumes	20	19.1 (10.2, 28.0)	262	29.4 (24.0, 34.8)	0.06	282	28.2 (23.2, 33.3)
Green, leafy vegetables	56	63.2 (49.3, 77.1)	496	57.8 (53.1, 62.6)	0.4	552	58.4 (53.7, 63.2)
Vitamin A‐rich fruits or vegetables	70	76.9 (64.4, 89.5)	700	77.4 (73.1, 81.7)	0.9	770	77.3 (73.3, 81.3)
Tea or Tibetan tea	42	37.2 (24.6, 49.9)	520	56.5 (51.0, 62.1)	<0.0001	562	54.4 (48.7, 60.2)
Minimum dietary diversity^o^	35	42.8 (30.6, 55.1)	414	48.3 (43.6, 53.1)	0.4	449	47.7 (43.0, 52.5)
Pica (%)	18	18.6 (9.4, 27.8)	127	12.3 (9.4, 15.2)	0.1	145	13.0 (10.1, 15.9)
Consumed micronutrient supplement^p^ (%)	0	‐	1	0.1 (0.0, 0.4)^c^	‐	1	0.1 (0.0, 0.4)^c^
Consumed iron‐folic acid^j^ (%)	1	0.3 (0.0, 1.0)^c^	12	1.1 (0.3, 1.9)	0.2	13	1.0 (0.3, 1.7)

Abbreviations: AGP, ɑ‐1 acid glycoprotein; BMI: body mass index; BMIZ: BMI‐for‐age *Z* scores; CI, confidence interval; CRP, C‐reactive protein; FAO: Food and Agriculture Organization; Hb: haemoglobin; NNMSS: Nepal National Micronutrient Status Survey; RBP, retinol binding protein; sTfR, transferrin receptor.

a Ns are unweighted. Values presented are geometric mean (95% CI) or percent (95% CI). All estimates account for weighting and complex sampling design. Anaemia defined as altitude‐ and smoking‐adjusted Hb <11.5 g/dL for boys 10–11 years, Hb <12.0 g/dL for boys 12–14 years and Hb <13.0 for boys 15–19 years (WHO^a^, 2017).

b *P* values calculated for Rao‐Scott chi square tests (categorical) and linear contrast tests (continuous).

c Interpret with caution. Estimates may be unstable due to small *n.*

d Other Terai ethnicities include Terai/Madhesi ethnicities not including Terai/Madhesi Brahmin/Chettri (Government of Nepal Central Bureau of Statistics, 2014).

e Water source based on self‐report. Unimproved water source defined as any source other than piped water, tubewell borehole, protected well or spring, stone tap, rainwater or bottle water (UNICEF & WHO, 2017).

f Household food insecurity was categorized according to the Household Food Insecurity Access Scale Indicator Guide (Coates, Swindale, & Billinksy, 2007).

g Haemoglobin adjusted for altitude and smoking (WHO^a^, 2017).

h Anaemia severity was classified as mild (adjusted Hb 11.0–11.4 g/dL for boys 10–11 years, Hb 11.0–11.9 g/dL for boys 12–14 years and Hb 11.0–12.9 g/dL for boys 15–19 years), moderate (adjusted Hb 8.0–10.9 g/dL) and severe (adjusted Hb <8.0 g/dL; WHO^a^, 2017).

i Underweight defined as BMI‐for‐age *Z* (BMIZ) < −2 *SD.* Normal weight defined as BMIZ ≥ −2 *SD* and BMIZ ≤1 *SD.* Overweight defined as BMIZ >1 *SD* (de Onis et al., 2007).

j During the 6 months preceding the survey.

k Biomarker was regression‐adjusted to a pooled country reference to adjust for inflammation, using CRP and AGP (ferritin) or AGP only (sTfR; Namaste et al., 2017).

l Iron deficiency by ferritin defined as inflammation‐adjusted serum ferritin <15.0 μg/L (WHO^a^, 2017).

m Iron deficiency by sTfR defined as inflammation‐adjusted serum sTfR>8.3 μg/L (WHO^a^, 2017).

n We defined vitamin A deficiency as RBP <0.64 μmol/L. To find the RBP cut‐point equivalent of retinol <0.70 μmol/L (WHO, 1996) among adolescents, we regressed RBP on retinol in an NNMSS subsample of 100 women 15–49 years for whom serum retinol was assessed using HPLC from the same blood draw as RBP.

o Minimum dietary diversity defined as intake from ≥5 of the 10 main food groups (grains, legumes, nuts, dairy, flesh foods, eggs, green leafy vegetables, vitamin A‐rich fruits and vegetables, other fruits, other vegetables) the day preceding the survey based on FAO recommendations for minimum dietary diversity for women of reproductive age (MDD‐W) (FAO and FHI 360, 2016).

p Reported micronutrient supplement intake includes multivitamin, vitamin A, iron tablets or syrup, folic acid and/or zinc tablets consumed the week preceding the survey.

Results of the bivariate analyses are presented in Table S2. Candidate predictors (*P* < 0.05 in bivariate analyses) in the multivariable model included both potentially modifiable factors (open defecation, earth floor, micronutrient status [sTfR, RBP] and dietary intake [animal‐flesh foods, tea]) and one nonmodifiable factor (ecological zone).

In the multivariable model, both iron status (ln sTfR in mg/L) and vitamin A status (ln RBP in μmol/L) were associated with anaemia odds (AOR 3.21, 95% CI [1.25, 8.23] and AOR 0.25, 95% CI [0.10, 0.65], respectively; Table 4). Having consumed flesh, organ or blood‐based foods during the day preceding the survey was associated with reduced odds of anaemia (AOR 0.57, 95% CI [0.33, 0.99]). Open defecation was associated with 2.36 times higher odds of anaemia (95% CI [1.15, 4.84]).

**Table 4:** Bivariate and multivariable logistic regression predicting anaemia among adolescent boys 10–19 years, Nepal National Micronutrient Status Survey, Nepal, 2016 (*n* = 967)^a^

Potential predictors of anaemia	Unadjusted odds ratio (95% CI)	Adjusted odds ratio (95% CI)	*P*
Ecological zone (ref. Terai)			
Mountain	0.24 (0.10, 0.58)	0.40 (0.14, 1.11)	0.08
Hill	0.50 (0.28, 0.91)	0.80 (0.41, 1.57)	0.5
Open defecation	3.71 (1.90, 7.24)	2.36 (1.15, 4.84)	0.02
Earth floor	1.79 (1.01, 3.17)	1.23 (0.67, 2.28)	0.5
Consumed flesh, organ or blood‐based foods	0.51 (0.28, 0.93)	0.57 (0.33, 0.99)	0.04
Consumed tea	0.46 (0.27, 0.76)	0.67 (0.38, 1.20)	0.1
Ln sTfR in mg/L^b^	4.63 (1.97, 10.85)	3.21 (1.25, 8.23)	0.02
Ln RBP in μmol/L	0.13 (0.05, 0.31)	0.25 (0.10, 0.65)	0.004

Abbreviations: Abbreviations: AGP, ɑ‐1 acid glycoprotein; CI, confidence interval; CRP, C‐ reactive protein; Hb: haemoglobin; RBP, retinol‐binding protein; sTfR, transferrin receptor.

a Estimates are unadjusted and adjusted odds ratios and 95% confidence intervals from bivariate and multivariable logistic regression, respectively. All analyses account for weighting and complex sampling design. We defined anaemia as altitude‐ and smoking‐adjusted Hb <11.5 g/dL for boys 10–11 years, Hb <12.0 g/dL for boys 12–14 years and Hb <13.0 for boys 15–19 years (WHO^a^, 2017). Candidate predictors were those where *P* < 0.05 in bivariate analyses.

b Biomarker was regression‐adjusted to a pooled country reference to adjust for inflammation, using CRP and AGP (Namaste et al., 2017).

## 4 DISCUSSION

We used nationally representative samples of adolescent boys and girls ages 10–19 years to explore anaemia in Nepal. In total, 10.9% of boys and 20.6% of girls had anaemia prevalence levels of mild and moderate public health significance, respectively, according to the WHO (WHO^a^, 2017). Among girls, residing in the Mountain or Hill ecological zones relative to the Terai, and better iron and vitamin A status were associated with reduced odds of anaemia, while older age and belonging to the Janajati ethnicity relative to the Muslim ethnicity were associated with increased odds of anaemia. Among boys, better vitamin A status and consumption of iron‐rich animal source foods were associated with reduced odds of anaemia and poorer iron (sTfR) status and household sanitation characteristics (open defecation) were associated with increased odds of anaemia. Evidence from this analysis suggests that some but not all of the burden of anaemia among adolescents in Nepal might be addressed through improved public health programming targeting micronutrient status, diet and sanitation.

Iron status was associated with anaemia in both boys and girls. Adolescents have increased nutritional demands during periods of rapid growth (Peduzzi, Concato, Kemper, Holford, & Feinstein, 1996) and, in resource constrained contexts, may have low access to nutrient‐rich diets (WHO, 2018). Girls are at additional risk for iron deficiency and anaemia due to the onset of menarche (WHO, 2018). Among adolescent girls, ferritin was inversely associated with anaemia odds. The burden of iron deficiency in adolescent girls was lower in our analyses compared with existing data. Thirty‐three percent of girls with anaemia had iron deficiency defined by ferritin in our analyses compared with 52% in the single study among adolescent girls aged 11–19 years in Dharan, Nepal, reporting both iron (ferritin) and anaemia status (Limbu et al., 2017). Five percent of boys in our analytic sample had iron deficiency defined by ferritin. To our knowledge, no other studies have reported the prevalence of iron deficiency among adolescent boys with anaemia in Nepal. Among boys, neither ferritin—the WHO recommended indicator to assess iron status in populations (WHO^a^, 2017)—nor iron deficiency defined by ferritin varied by anaemia status in bivariate analyses; however, sTfR was associated with anaemia in both bivariate and multivariable analyses, suggesting that iron may have some contribution to anaemia burden among adolescent boys in Nepal. Further, having consumed iron‐rich flesh, organ or blood‐based foods the day preceding the survey was associated with 43% lower odds of anaemia among boys.

Vitamin A status (RBP) was associated with reduced odds of anaemia in both girls and boys in our study. Vitamin A is required for erythropoiesis and mobilization of iron stores (WHO^a^, 2017). Although we were unable to estimate total vitamin A intake, reported consumption of food sources high in vitamin A the day preceding the survey did not vary by anaemia status for either boys or girls.

Strategies to improve iron status and vitamin A status among adolescent boys and girls might help reduce anaemia in Nepal. The WHO recommends intermittent IFA supplementation to all children aged 10–12 years and menstruating adolescent girls where anaemia prevalence is ≥20% (WHO^a^, 2011; WHO^b^, 2011). In 2016, the MoHP in Nepal began scaling up a program to provide weekly IFA supplementation to girls 10–19 years (Ministry of Health et al., 2018); however, reported intake of IFA during the 6 months preceding the survey among girls was low (<2%). The WHO also recommends fortification to address nutritional causes of anaemia (WHO, 2009). Although Nepal mandates fortification of industrially produced wheat flour with iron, vitamin A and folic acid and industrially produced vegetable ghee with vitamin A, purchasing and consumption levels were low. Less than half of households (45.4%) purchased potentially fortified wheat flour from large‐scale producers (Ministry of Health et al., 2018), and <3% of adolescents reported consuming vegetable ghee the day preceding the survey (data not presented). WHO/FAO guidance on food fortification recommends appropriate design and monitoring to support quality industrial food fortification (Dary & Hurrell, 2006); it is also critical to consider the mix of public health interventions to best reach the population. Programs aimed at promoting nutrient‐rich diets and enhanced bioavailability of micronutrients through food preparation and processing could help improve iron and vitamin A status (WHO^a^, 2017). Tools exist to model intervention scenarios with different interventions and potential impact (Brown, Engle‐Stone, Kagin, Rettig, & Vosti, 2015), which may be useful to countries as they review policies and their mix and reach of programs.

Age was associated with anaemia among girls but not boys. Older age in girls was associated with higher anaemia odds in the multivariable model. Findings concerning age and anaemia in Nepal have been equivocal. Kanodia et al. reported higher anaemia prevalence among girls in early adolescence (10–13 years) relative to girls in middle (14–15 years) and late (16–19 years) adolescence in Dharan, Nepal (Kanodia et al., 2016), while three other studies in Dharan, urban Kathmandu and Morang District reported no differences by age (Limbu et al., 2017; Shah & Gupta, 2002; Tiwari & Seshadri, 2000), though these four studies only assessed bivariate relationships between age and anaemia. The single study with multivariable analyses reported 75% higher odds of anaemia (95% CI [1.44, 2.13]) among adolescent boys and girls 15–19 years relative to adolescents 10–14 years in nationally representative samples (Chalise et al., 2018). Lower odds of anaemia among younger girls in our study might be explained by shorter reproductive history (WHO^a^, 2017); however, no data were available on onset of menses. Even though age is nonmodifiable, information about anaemia patterning might be used to target anaemia prevention and control initiatives to older girls in Nepal.

Anaemia was patterned by ecological zone among girls. Girls residing in the Mountain or Hill ecological zones had lower odds of anaemia relative to those residing in the Terai ecological zone. Similarly, Chalise et al. reported 80% higher odds of anaemia among adolescent boys and girls 10–19 years residing in the Terai zone relative to the Mountain zone in a nationally representative sample (Chalise et al., 2018). Geographic patterning of congenital blood disorders might explain regional differences in anaemia; however, residing in the Mountain or Hill ecological zones was associated with reduced odds of anaemia among nonpregnant women 15–49 years in Nepal after adjustment for blood disorders, suggesting that regional differences in anaemia could be due to other factors (Ford, Paudyal, Pokharel, et al., 2018). Chronic exposure to arsenic via contaminated groundwater might explain regional differences in anaemia. Studies in the Terai have documented groundwater arsenic levels exceeding the upper limit for drinking water per the WHO guidelines (>10 μg/L; WHO^b^, 2017; Pokhrel, Bhandari, & Viraraghavan, 2009). Arsenic exposure can lead to anaemia through increased erythrocytes hemolysis (Mahmud, Foller, & Lang, 2008) and reduced heme metabolism (Hernandez‐Zavala et al., 1999). Future research might explore arsenic exposure and anaemia.

Among girls, the Janajati ethnicity had higher odds of anaemia relative to the Muslim ethnicity. Two studies, one nationally representative and one from Kathmandu, examined anaemia by ethnicity and reported no significant differences (Chalise et al., 2018; Tiwari & Seshadri, 2000); however, classifications of ethnicity varied across studies and are thus not directly comparable to our findings. Ethnic differences might be explained in part by congenital blood disorders, dietary practices or other practices not captured by this survey.

Among boys, anaemia status varied by household sanitation characteristics. In bivariate analyses, a higher percentage of boys with anaemia resided in a house without a toilet facility and in homes with a dirt floor relative to boys without anaemia. Open defecation was associated with more than double the odds of anaemia in the multivariable model; however, dirt floor was no longer significant after adjusting for other variables. We had similar findings among girls for open defecation in the bivariate analyses; however, this indicator was not significant in the multivariable model. Lack of toilet facility and dirt floors can expose household members to faecal matter, worms, protozoa and other parasites (WHO^a^, 2017), leading to infection. Similarly, a nationally representative study of adolescent boys and girls aged 10–19 years in Nepal found higher odds of anaemia among those who reported walking barefoot relative to those who wore shoes (Chalise et al., 2018). Although the NNMSS collected data on recent morbidity and measured biomarkers of inflammation, we did not have biological data on soil transmitted helminth infection among adolescents. Some evidence suggests that community‐level sanitation variables could play a role in health (Headey, Hoddinott, & Park, 2017). One study in Ecuador reported that community‐level sanitation coverage was a stronger predictor of child stunting than the household sanitation status (Fuller, Villamor, Cevallos, Trostle, & Eisenberg, 2016). To our knowledge, no studies have explored the role of community‐level sanitation on anaemia specifically. Future research might explore the role of community‐level sanitation on anaemia in Nepal.

### 4.1 Strengths and limitations

To our knowledge, this analysis is the first to examine a wide range of known potential causes of anaemia in a nationally representative sample of adolescent boys and girls in Nepal. We used data on multiple potential biological causes of anaemia, not often included from large‐scale, population‐based surveys, such as multiple biomarkers of micronutrient status and inflammation. Due to the cross‐sectional study design, we were unable to establish causality between candidate predictors and anaemia status; however, our study contributes to the limited evidence base in this understudied population group. Among adolescents, the NNMSS did not collect data on some micronutrients for which deficiency could lead to anaemia, such B_12_, or on other potentially important biomarkers (WHO^a^, 2017). RBP is not the WHO‐recommended indicator to assess vitamin A status (WHO, 1996). Dietary recall questions were limited in scope, and we used a dietary diversity tool created for women of reproductive age. There is no internationally accepted tool to measure adolescent diets. Although interview could have introduced recall and social desirability bias for reported dietary and micronutrient intake and household food insecurity, it was not likely to be differential by anaemia status because adolescents completed the survey questionnaire prior to having Hb assessed. Sample sizes were calculated to estimate national‐level prevalence of anaemia and iron deficiency; thus, we may have been underpowered to detect risk factors with small effect sizes in multivariable models. Finally, adolescents excluded from the analyses were on average older than those who were included, potentially reducing generalizability of the findings to older adolescents.

## 5 CONCLUSION

More than 1 in 10 boys and 1 in 5 girls had anaemia. Our findings suggest that strategies to improve iron status, vitamin A status, diet and sanitation could potentially reduce anaemia among adolescents in Nepal. Nonmodifiable factors such as age, ethnicity and ecological zone could explain differential success in reducing anaemia and might help provide context to program monitoring and evaluation and inform program strategies.

## CONFLICTS OF INTEREST

The authors declare that they have no conflicts of interest.

## CONTRIBUTIONS

MEJ, RDW, ZM, RFA, NP, SC, SR, KRP, RPB and NJ designed the research. NJ, NP, SC, DA, SR, KRP and RPB conducted the research. NJ performed the initial database cleaning. NDF performed the statistical analyses and wrote the paper. NDF, NP, NJ, RPB, KRP, RDW, SC, SR, ZM, RFA, DA and MEJ edited subsequent drafts. NDF had primary responsibility for the final content. All authors have read and approved the manuscript.

## Supporting information


**Table S1.** Selected Sociodemographic and Health Characteristics of Adolescent Girls and Boys 10–19 Years, by Inclusion in the Analytic Sample, Among Adolescents who Consented to Participate in the Nepal National Micronutrient Status Survey, Nepal, 2016 (*n* = 1,850 for Girls and *n* = 1,025 for Boys) ^1^

**Table S2.** Binomial Logistic Regression Predicting Anemia Among Non‐Pregnant Adolescent Girls and Boys 10–19 Years, Nepal National Micronutrient Status Survey, Nepal, 2016 (*n* = 1,680 for Girls and *n* = 967 for Boys) ^1^

## References

[mcn13013-bib-0002] Baral, K. P. , & Onta, S. R. (2009). Prevalence of anemia amongst adolescents in Nepal: A community based study in rural and urban areas of Morang District. Nepal Medical College Journal, 11(3), 179–182. 20334065

[mcn13013-bib-0003] Brown, K. H. , Engle‐Stone, R. , Kagin, J. , Rettig, E. , & Vosti, S. A. (2015). Use of optimization modeling for selecting national micronutrient intervention strategies: An example based on potential programs for control of vitamin A deficiency in Cameroon. Food and nutrition bulletin, 36(3_suppl), S141–S148. 26283708 10.1177/0379572115599325

[mcn13013-bib-0004] Chalise, B. , Aryal, K. K. , Mehta, R. K. , Dhimal, M. , Sapkota, F. , Mehata, S. , … Sawyer, S. (2018 Dec 14). Prevalence and correlates of anemia among adolescents in Nepal: Findings from a nationally representative cross‐sectional survey. PLoS ONE, 13(12), e0208878. 10.1371/journal.pone.0208878 30551124 PMC6294609

[mcn13013-bib-0005] Coates, J. , Swindale, A. , & Billinsky, P. (2007). Household Food Insecurity Access Scale (HFIAS) for measurement of household food access: Indicator guide (v. 3). Washington, D.C.: FHI 360/FANTA.

[mcn13013-bib-0006] Dary, O. , & Hurrell, R. (2006). Guidelines on food fortification with micronutrients. Geneva: Switzerland World Health Organization, Food and Agricultural Organization of the United Nations.

[mcn13013-bib-0007] Delisle, H. , & World Health Organization . (2005). Nutrition in adolescence issues and challenges for the health sector: Issues in adolescent health and development. Geneva: Switzerland, World Health Organization.

[mcn13013-bib-0008] Dubey, R. K. , Padmavathi, P. , Jayan, A. , Gautam, N. , Neupane, Y. , & Sinha, A. K. (2013). Prevalence of Anemia Amongst Adolescent Females in South Western Nepal. Pharma Innovation., 2(7), 70–75.

[mcn13013-bib-0009] Erhardt, J. G. , Estes, J. E. , Pfieffer, C. M. , Biesalski, H. K. , & Craft, N. E. (2004 Nov). Combined measurement of ferritin, soluble transferrin receptor, retinol binding protein, and C‐reactive protein by an inexpensive, sensitive, and simple sandwich enzyme‐linked immunosorbent assay technique. The Journal of Nutrition, 134(11), 3127–3132. 10.1093/jn/134.11.3127 15514286

[mcn13013-bib-0010] Ezzati, M. , Lopez, A. D. , Rodgers, A. A. , & Murray, C. J. L. (2004). Comparative quantification of health risks: Global and regional burden of disease attributable to selected major risk factors. Geneva, Switzerland: WHO.

[mcn13013-bib-0011] FAO and FHI 360 . (2016). Minimum dietary diversity for women: A guide to measurement. Rome, Italy: Food and Agriculture Organization of the United Nations.

[mcn13013-bib-0012] Ford, ND , Paudyal N , Pokharel R , et al. Determinants of anemia in a nationally representative sample of nonpregnant women of reproductive age in Nepal (OR08–06). Current Developments in Nutrition, 1 November 2018;11:(2). 10.1093/cdn/nzy039

[mcn13013-bib-0013] Fuller, J. A. , Villamor, E. , Cevallos, W. , Trostle, J. , & Eisenberg, J. N. (2016 Apr 1). I get height with a little help from my friends: Herd protection from sanitation on child growth in rural Ecuador. International Journal of Epidemiology., 45(2), 460–469. 10.1093/ije/dyv368 26936912 PMC5841884

[mcn13013-bib-0014] Government of Nepal Central Bureau of Statistics . (2014). Statistical pocketbook of Nepal 2014. Katmandhu, Nepal: Government of Nepal.

[mcn13013-bib-0015] Headey, D. , Hoddinott, J. , & Park, S. (2017 Jun 1). Accounting for nutritional changes in six success stories: A regression‐decomposition approach. Global Food Security., 13, 12–20.

[mcn13013-bib-0016] Hernandez‐Zavala, A. , Del Razo, L. M. , Garcia‐Vargas, G. G. , Aguilar, C. , Borja, V. H. , Albores, A. , & Cebrián, M. E. (1999 Jun). Altered activity of heme biosynthesis pathway enzymes in individuals chronically exposed to arsenic in Mexico. Arch Toxicol, 2(73), 90–95. 10.1007/s002040050592 10350189

[mcn13013-bib-0017] Institute of Medicine, Food and Nutrition Board . (1998). Dietary reference intakes: Thiamin, riboflavin, niacin, vitamin B6, folate, vitamin B12, pantothenic acid, biotin, and choline. Washington (DC): National Academy Press. 23193625

[mcn13013-bib-0018] Kanodia, P. , Bhatta, M. , Sing, R. R. , Bhatta, N. K. , & Shah, G. S. (2016). A study of anemia among adolescent girls in eastern part of Nepal. Journal of College of Medical Sciences‐Nepal., 12(1), 19–22.

[mcn13013-bib-0019] Limbu, N. , Thakur, D. , Das, B. K. L. , Choudhary, L. B. , Pradhan, A. , & Baral, D. (2017). Prevalence of anemia and iron deficiency in adolescent school girls in Dharan. Nepal. Asian Journal of Medical Science., 8(5), 22–26.

[mcn13013-bib-0020] Mahmud, H. , Foller, M. , & Lang, F. (2008 Jul). Arsenic‐induced suicidal erythrocyte death. Archives of Toxicology, 83(2), 107–113. 10.1007/s00204-008-0338-2 18636241

[mcn13013-bib-0021] Merrill, R. D. , Shamim, A. A. , Ali, H. , Labrique, A. B. , Schulze, K. , Christian, P. , & West, K. P. (2012). High prevalence of anemia with lack of iron deficiency among women in rural Bangladesh: A role for thalassemia and iron in groundwater. Asia Pacific Journal of Clinical Nutrition., 21(3), 416–424. 22705433

[mcn13013-bib-0022] Ministry of Health and Population ‐ MOHP/Nepal, New ERA/Nepal, and ICF International . (2012). Nepal demographic and health survey 2011. Kathmandu, Nepal: MOHP/Nepal, New ERA/Nepal, and ICF International.

[mcn13013-bib-0023] Ministry of Health Nepal, New ERA, ICF . (2017). In Nepal demographic and health survey 2016. Kathmandu, Nepal: Ministry of Health Nepal.

[mcn13013-bib-0024] Ministry of Health, New ERA, UNICEF, EU, USAID, CDC . (2018). Nepal national micronutrient status survey‐2016. Kathmandu, Nepal: Ministry of Health, Nepal.

[mcn13013-bib-0025] Namaste, S. M. L. , Aaron, G. J. , Varadhan, R. , Peerson, J. M. , & Suchdev, P. S. (2017 Jul). Methodological approach for the Biomarkers Reflecting Inflammation and Nutritional Determinants of Anemia (BRINDA) project. The American Journal of Clinical Nutrition, 106(Suppl 1), 333S–347S. 10.3945/ajcn.116.142273 28615254 PMC5490643

[mcn13013-bib-0026] de Onis, M. , Onyango, A. W. , Borghi, E. , Siyam, A. , Nishida, C. , & Siekmann, J. (2007). Development of a WHO growth reference for school‐aged children and adolescents. Bulletin of the World Health Organization, 85, 660–667. 10.2471/blt.07.043497 18026621 PMC2636412

[mcn13013-bib-0027] Peduzzi, P. , Concato, J. , Kemper, E. , Holford, T. R. , & Feinstein, A. R. (1996 Dec). A simulation study of the number of events per variable in logistic regression analysis. Journal of Clinical Epidemiology, 49(12), 1373–1379. 10.1016/s0895-4356(96)00236-3 8970487

[mcn13013-bib-0028] Pfeiffer, C. M. , Zhang, M. , Lacher, D. A. , Molloy, A. M. , Tamura, T. , Yetley, E. A. , … Johnson, C. L. (2011). Comparison of serum and red blood cell folate microbiologic assays for national population surveys. The Journal of Nutrition, 141(7), 1402–1409. 10.3945/jn.111.141515 21613453 PMC3113292

[mcn13013-bib-0029] Pokhrel, D. , Bhandari, B. S. , & Viraraghavan, T. (2009). Arsenic contamination in the Terai region of Nepal: An overview of health concerns and treatment options. Environmental International., 35(1), 157–161. 10.1016/j.envint.2008.06.003 18723222

[mcn13013-bib-0030] Sawyer SM , Afifi RA , Bearinger LH , Blakemore SJ , Dick B , Ezeh AC , Patton GC . Adolescence: A foundation for future health. Lancet. 28 Apr 2012;379(9826):1630–40. 22538178 10.1016/S0140-6736(12)60072-5

[mcn13013-bib-0031] Shah, B. K. , & Gupta, P. (2002). Anemia in adolescent girls: A preliminary report from semi‐urban Nepal. Indian Pediatrics., 39, 1126–1130. 12522274

[mcn13013-bib-0032] Sinha, A. K. , Karki, G. M. S. , & Karna, K. K. (2012). Prevalence of anemia amongst adolescents in Biratnagar, Morang Dist. Nepal. International Journal of Pharmaceutical and Biological Archive., 3(5), 1077–1081.

[mcn13013-bib-0033] Tiwari, K. , & Seshadri, S. (2000). The prevalence of anemia and morbidity profile among school going adolescent girls of urban Kathmandu. Nepal. J Nepal Med Assoc., 39, 319–325.

[mcn13013-bib-0034] WHO . (1996). Indicators for assessing vitamin A deficiency and their application in monitoring and evaluating intervention programmes. Geneva, Switzerland: World Health Organization.

[mcn13013-bib-0035] WHO . (2009). Recommendations on wheat and maize flour fortification meeting report: Interim consensus statement. WHO/NMH/NHD/MNM/09.1. Geneva, Switzerland: World Health Organization. 24809114

[mcn13013-bib-0036] WHO . (2015). Global health estimates 2015: DALYs by cause, age, sex, by country and by region, 2000–2015. Geneva, Switzerland: World Health Organization.

[mcn13013-bib-0037] WHO . (2017a). Nutritional anaemias: Tools for effective prevention and control. Geneva: World Health Organization. License: CC BY‐NC‐SA 3.0 IGO

[mcn13013-bib-0038] WHO . (2017b). Guidelines for drinking‐water quality: Fourth edition incorporating the first addendum. Geneva, Switzerland: World Health Organization. License CC BY‐NC‐SA 3.0 IGO 28759192

[mcn13013-bib-0039] WHO . (2018). Guideline: Implementing effective actions for improving adolescent nutrition. Geneva, Switzerland: World Health Organization. License CC BY‐NC‐SA 2.0 IGO

[mcn13013-bib-0040] WHO and UNICEF . (2017). Progress on drinking water, sanitation and hygiene: 2017 Update and SDG baselines. Geneva, Switzerland: World Health Organization and the United Nations Children's Fund.

[mcn13013-bib-0041] WHO Regional Office for South‐East Asia . (2011). Prevention of iron deficiency anaemia in adolescents: Role of weekly iron and folic acid supplementation. New Dehli, India: World Health Organization Regional Office for South‐East Asia.

[mcn13013-bib-0042] WHOa . (2011). Guideline: Intermittent iron supplementation in preschool and school‐age children. Geneva: World Health Organization. 24479203

[mcn13013-bib-0043] WHOb . (2011). Guideline: Intermittent iron and folic acid supplementation in menstruating women. Geneva: World Health Organization. 24479204

[mcn13013-bib-0044] Wieringa, F. , Dahl, M. , Chamnan, C. , Poirot, E. , Kuong, K. , Sophonneary, P. , … Dijkhuizen, M. (2016). The high prevalence of anemia in Cambodian children and women cannot be satisfactorily explained by nutritional deficiencies or hemoglobin disorders. Nutrients, 8(6), 348. 10.3390/nu8060348 PMC4924189 27338454

