# Supplementary material for: Factors associated with anaemia among adolescent boys and girls 10–19 years old in Nepal
Source: Matern Child Nutr. 2020 Apr 27;18(Suppl 1):e13013. doi: 10.1111/mcn.13013 (PMC8770652; doi:10.1111/mcn.13013)
Supplement: Supplementary file 1 — Table S1. Selected Sociodemographic and Health Characteristics of Adolescent Girls and Boys 10–19 Years, by Inclusion in the Analytic Sample, Among Adolescents who Consented to Participate in the Nepal National Micronutrient Status Survey, Nepal, 2016 (n = 1,850 for Girls and n = 1,025 for Boys) 1 [file MCN-18-e13013-s001.docx]

**Supplemental Table 1.** Selected Sociodemographic and Health Characteristics of Adolescent Girls and Boys 10-19 Years, by Inclusion in the Analytic Sample, Among Adolescents who Consented to Participate in the Nepal National Micronutrient Status Survey, Nepal, 2016 (*n*=1,850 for Girls and *n*=1,025 for Boys) ^1^

|  | Adolescent Girls | | | | | Adolescent Boys | | | | |
| --- | --- | --- | --- | --- | --- | --- | --- | --- | --- | --- |
|  | Included in Analytic Sample  (*n*=1,680, 90.8%) | | Excluded from Analytic Sample  (*n*=170, 9.2%) | |  | Included in Analytic Sample  (*n*=967, 94.5%) | | Excluded from Analytic Sample  (*n*=58, 5.5%) | |  |
|  | *n* |  | *n* |  | *P* ^2^ | *n* |  | *n* |  | *P* ^2^ |
| Sociodemographic and Health Characteristics |  |  |  |  |  |  |  |  |  |  |
| Age, years | 1680 | 13.9 (13.8,14.1) | 170 | 17.9 (17.4,18.4) | <0.0001 | 967 | 13.9 (13.7,14.1) | 58 | 17.0 (16.1,18.0) | <0.0001 |
| Married/cohabitating, % | 123 | 7.7 (5.9,9.5) | 64 | 38.9 (29.4,48.3) | <0.0001 | 24 | 2.4 (1.3,3.6) | 5 | 8.7 (0.0,18.7)^4^ | 0.01 |
| Location, % |  |  |  |  | 0.06 |  |  |  |  | 0.3 |
| Rural | 1491 | 90.3 (85.5,95.2) | 144 | 85.4 (76.1,94.7) |  | 830 | 85.9 (79.1,92.6) | 52 | 91.1 (82.9,99.3) |  |
| Urban | 189 | 9.7 (4.8,14.5) | 26 | 14.6 (5.3,23.9) |  | 137 | 14.1 (7.4,20.9) | 6 | 8.9 (0.7,17.1) |  |
| Ecological zone, % |  |  |  |  | 0.7 |  |  |  |  | 0.7 |
| Mountain | 265 | 7.6 (6.2,8.9) | 24 | 6.0 (3.0,9.0) |  | 148 | 6.8 (5.9,7.7) | 9 | 7.3 (2.2,12.4)^4^ |  |
| Hill | 707 | 43.5 (39.5,47.4) | 71 | 46.7 (37.4,56.1) |  | 410 | 41.6 (37.8,45.3) | 25 | 46.5 (32.1,60.8) |  |
| Terai | 708 | 49.0 (45.1,52.8) | 75 | 47.2 (37.8,56.6) |  | 409 | 51.6 (47.9,55.4) | 24 | 46.2 (31.5,61.0) |  |
| Household wealth tertile |  |  |  |  | 0.002 |  |  |  |  | 0.7 |
| Poorest | 638 | 33.0 (28.2,37.9) | 46 | 20.9 (13.9,28.0) |  | 317 | 26.0 (21.2,30.9) | 18 | 24.1 (11.1,37.1) |  |
| Middle | 559 | 35.3 (31.6,39) | 54 | 34.0 (24.6,43.3) |  | 335 | 36.6 (31.5,41.6) | 22 | 42.4 (27.5,57.3) |  |
| Wealthiest | 483 | 31.6 (26.1,37.1) | 70 | 45.1 (34.5,55.7) |  | 315 | 37.4 (30.9,43.9) | 18 | 33.5 (19.2,47.9) |  |
| Ethnicity, % |  |  |  |  | 0.4 |  |  |  |  | 0.9 |
| Brahmin/Chettri | 651 | 33.5 (27.6,39.4) | 54 | 28.4 (19.3,37.5) |  | 409 | 35.1 (29.4,40.9) | 27 | 44.7 (29.0,60.4) |  |
| Dalit | 291 | 16.5 (12.3,20.8) | 32 | 14.7 (7.7,21.7) |  | 149 | 14.4 (10.0,18.7) | 10 | 14.9 (3.2,26.6) |  |
| Janajati | 542 | 32.9 (26.2,39.7) | 60 | 33.9 (24.0,43.7) |  | 285 | 29.6 (23.6,35.6) | 16 | 28.1 (13.5,42.6) |  |
| Other Terai ethnicities ^3^ | 113 | 11.5 (6.9,16.2) | 12 | 14.2 (5.4,23.0) |  | 68 | 13.5 (8.4,18.7) | 2 | 6.1 (0.0,14.7)^4^ |  |
| Newar | 49 | 3.2 (1.6,4.9) | 9 | 6.5 (1.9,11.1) |  | 34 | 4.3 (1.6,6.9) | 3 | 6.2 (0.0,14.0)^4^ |  |
| Muslim | 34 | 2.3 (0.6,4.0) | 3 | 2.3 (0.0,5.2) |  | 22 | 3.1 (1.0,5.2) | 0 | - |  |
| Never attended school, % | 62 | 5.6 (2.7,8.5) | 5 | 4.7 (0.8,9.3)^4^ | 0.8 | 14 | 2.0 (0.5,3.5) | 0 | - | 0.9 |

^1^ Ns are unweighted. Values presented are percent (95% CI). All estimates account for weighting and complex sampling design.

^2^ P values calculated for Rao-scott chi square tests (categorical).

^3^ Other Terai ethnicities include Terai/Madhesi ethnicities not including Terai/Madhesi Brahmin/Chettri.

^4^ Interpret with caution. Estimates may be unstable due to small n.

Abbreviations: CI, confidence interval.
