# Supplementary material for: Factors associated with anaemia among adolescent boys and girls 10–19 years old in Nepal
Source: Matern Child Nutr. 2020 Apr 27;18(Suppl 1):e13013. doi: 10.1111/mcn.13013 (PMC8770652; doi:10.1111/mcn.13013)
Supplement: Supplementary file 2 — Table S2. Binomial Logistic Regression Predicting Anemia Among Non‐Pregnant Adolescent Girls and Boys 10–19 Years, Nepal National Micronutrient Status Survey, Nepal, 2016 (n = 1,680 for Girls and n = 967 for Boys) 1 [file MCN-18-e13013-s002.docx]

**Supplemental Table 2.** Binomial Logistic Regression Predicting Anemia Among Non-Pregnant Adolescent Girls and Boys 10-19 Years, Nepal National Micronutrient Status Survey, Nepal, 2016 (*n*=1,680 for Girls and *n*=967 for Boys) ^1^

|  | Adolescent Girls  (*n*=1,680) | | Adolescent Boys  (*n*=967) | |
| --- | --- | --- | --- | --- |
|  | Unadjusted Odds Ratio (95% CI) | *P^2^* | Unadjusted Odds Ratio (95% CI) | *P^2^* |
| Sociodemographic characteristics |  |  |  |  |
| Age, years | 1.11 (1.06, 1.16) | <0.0001 | 1.03 (0.93, 1.13) | 0.6 |
| Lactating | 1.60 (0.76, 3.38) | 0.2 | - | - |
| Gave birth in last 5 years | 1.52 (0.72, 3.19) | 0.3 | - | - |
| Married/cohabitating | 1.34 (0.81, 2.21) | 0.2 | 2.40 (0.60, 9.64) | 0.2 |
| Urban residence | 1.11 (0.66, 1.85) | 0.7 | 0.54 (0.24, 1.21) | 0.1 |
| *Ecological zone (ref. Terai)* |  | <0.0001 |  | 0.002 |
| Mountain | 0.24 (0.12, 0.46) |  | 0.24 (0.10, 0.58) |  |
| Hill | 0.39 (0.24, 0.63) |  | 0.50 (0.28, 0.91) |  |
| *Household wealth tertile (ref. wealthiest)* |  | 0.2 |  | 0.2 |
| Poorest | 0.72 (0.46, 1.13) |  | 1.80 (0.87, 3.75) |  |
| Middle | 0.97 (0.68, 1.38) |  | 1.69 (0.83, 3.45) |  |
| *Ethnicity (ref. Muslim)* |  | 0.02 |  | 0.2 |
| Dalit | 0.81 (0.28, 2.30) |  | 1.62 (0.26, 10.14) |  |
| Janajati | 1.14 (0.39, 3.38) |  | 3.61 (0.62, 21.03) |  |
| Other Terai ethnicities ^3^ | 1.43 (0.44, 4.67) |  | 3.58 (0.60, 21.15) |  |
| Newar | 0.36 (0.05, 2.76) |  | 0.76 (0.05, 11.63) |  |
| Brahmin/Chettri | 0.59 (0.20, 1.81) |  | 1.80 (0.31, 10.38) |  |
| Never attended school | 1.58 (0.82, 3.04) | 0.2 | 2.17 (0.59, 7.98) | 0.2 |
| Unimproved water source ^4^ | 0.67 (0.21, 2.14) | 0.5 | 1.28 (0.42, 3.97) | 0.6 |
| Open defecation | 2.03 (1.26, 3.28) | 0.004 | 3.71 (1.90, 7.24) | 0.0002 |
| Earth floor | 0.93 (0.64, 1.36) | 0.7 | 1.79 (1.01, 3.17) | 0.04 |
| Household food insecurity ^5^*(ref. food secure)* |  | 0.3 |  | 0.2 |
| Mild food insecurity | 1.19 (0.74, 1.70) |  | 1.11 (0.59, 2.12) |  |
| Moderate food insecurity | 0.61 (0.30, 1.23) |  | 1.76 (0.51, 6.10) |  |
| Severe food insecurity | 0.93 (0.59, 1.46) |  | 0.37 (0.11, 1.20) |  |
| Health characteristics |  |  |  |  |
| *Anthropometry ^6^ (ref. normal weight)* |  | 0.9 |  | 0.07 |
| Underweight | 0.90 (0.59, 1.39) |  | 1.59 (0.90, 2.78) |  |
| Overweight/obesity | 0.97 (0.42, 2.21) |  | 0.20 (0.02, 1.64) |  |
| *Two-week morbidity recall* |  |  |  |  |
| Fever | 1.29 (0.82, 2.03) | 0.3 | 1.42 (0.67, 3.02) | 0.3 |
| Cough | 0.97 (0.65, 1.46) | 0.9 | 0.45 (0.15, 1.34) | 0.1 |
| Diarrhea | 0.81 (0.49, 1.35) | 0.4 | 1.60 (0.58, 4.41) | 0.4 |
| CRP, mg/L | 1.00 (0.91, 1.10) | 0.9 | 1.08 (0.84, 1.38) | 0.6 |
| AGP, g/L | 1.34 (0.89, 2.01) | 0.2 | 1.27 (0.63, 2.55) | 0.5 |
| Helicobacter pylori | 1.05 (0.71, 1.54) | 0.8 | 1.03 (0.53, 2.03) | 0.9 |
| Received deworming ^7^ | 0.91 (0.66, 1.26) | 0.6 | 1.12 (0.63, 1.99) | 0.7 |
|  |  |  |  |  |
| Micronutrient status |  |  |  |  |
| Serum ferritin ^8^, μg/L | 0.51 (0.40, 0.66) | <0.0001 | 1.12 (0.57, 2.19) | 0.7 |
| Serum sTfR^8^, mg/L | 5.64 (3.63, 8.76) | <0.0001 | 4.63 (1.97, 10.85) | 0.0005 |
| Serum RBP, μmol/L | 0.12 (0.07, 0.23) | <0.0001 | 0.13 (0.05, 0.31) | <0.0001 |
| RBC folate, nmol/L | 0.83 (0.58, 1.19) | 0.3 | - | - |
|  |  |  |  |  |
| Dietary and supplement intake |  |  |  |  |
| *Prior day food consumption* |  |  |  |  |
| Flesh, organ, or blood-based foods | 1.08 (0.75, 1.57) | 0.7 | 0.51 (0.28, 0.93) | 0.03 |
| Legumes | 1.13 (0.84, 1.52) | 0.4 | 1.76 (0.95, 3.26) | 0.07 |
| Green, leafy vegetables | 1.14 (0.85, 1.54) | 0.4 | 0.80 (0.44, 1.45) | 0.4 |
| Vitamin A-rich fruits or vegetables | 1.27 (0.77, 2.09) | 0.3 | 1.03 (0.48, 2.18) | 0.9 |
| Tea or Tibetan tea | 0.85 (0.59, 1.22) | 0.4 | 0.46 (0.27, 0.76) | 0.003 |
| Minimum dietary diversity ^9^ | 1.04 (0.72, 1.51) | 0.8 | 0.80 (0.49, 1.32) | 0.4 |
| Pica | 1.04 (0.68, 1.60) | 0.8 | 1.63 (0.86, 1.08) | 0.1 |
| Consumed micronutrient supplement ^10^ | 2.19 (0.91, 5.26) | 0.08 | -^10^ | - |
| Consumed iron-folic acid ^7^ | 2.97 (1.19, 7.44) | 0.02 | 0.30 (0.04, 2.44) | 0.2 |

^1^ Estimates are unadjusted odds ratios and 95% confidence intervals from bivariate logistic regression. All analyses account for weighting and complex sampling design. We defined anemia as altitude- and smoking-adjusted Hb <11.5 g/dL for boys and girls 10-11 years, Hb <12.0 g/dL for boys 12-14 years and girls 12-19 years, and Hb <13.0 for boys 15-19 years (WHO^a^ 2017).

^2^ Biomarker was regression-adjusted to a pooled country reference to adjust for inflammation, using CRP and AGP (Namaste et al 2017).

^3^ Other Terai ethnicities include Terai/Madhesi ethnicities not including Terai/Madhesi Brahmin/Chettri (Government of Nepal Central Bureau of Statistics 2014).

^4^ Water source based on self-report. Unimproved water source defined as any source other than piped water, tubewell borehole, protected well or spring, stone tap, rainwater, or bottle water (UNICEF and WHO 2017).

^5^Household food insecurity was categorized according to the Household Food Insecurity Access Scale Indicator Guide (Coates, Swindale, and Billinksy 2007).

^6^Underweight defined as BMIZ <-2 SD. Normal weight defined as BMIZ ≥ -2 SD and BMIZ ≤ 1 SD. Overweight defined as BMIZ >1 SD (deOnis et al 2007).

^7^During the six months preceding the survey.

^8^Biomarker was regression-adjusted to a pooled country reference to adjust for inflammation, using CRP and AGP (ferritin) or AGP only (sTfR) (Namaste et al. 2017).

^9^Minimum dietary diversity defined as intake from ≥5 of the 10 main food groups (grains, legumes, nuts, dairy, flesh foods, eggs, green leafy vegetables, vitamin A-rich fruits and vegetables, other fruits, other vegetables) the day preceding the survey based on FAO recommendations for minimum dietary diversity for women (FAO and FHI 360 2016).

^10^Reported micronutrient supplement intake includes multivitamin, vitamin A, iron tablets or syrup, folic acid, and/or zinc tablets consumed the week preceding the survey.

^11^ Model did not converge.

Abbreviations: AGP, ɑ-1 acid glycoprotein; CI, confidence interval; CRP, C-reactive protein; RBC, red blood cell; RBP, retinol-binding protein.
